# Mechanical and Optical Properties of Cr_2_O_3_ Thin Films Grown by Atomic Layer Deposition Method Using Cr(thd)_3_ and Ozone

**DOI:** 10.3390/nano13192702

**Published:** 2023-10-04

**Authors:** Mahtab Salari Mehr, Lauri Aarik, Taivo Jõgiaas, Aarne Kasikov, Elyad Damerchi, Hugo Mändar

**Affiliations:** 1 Institute of Physics, University of Tartu, W. Ostwald Str. 1, 50411 Tartu, Estonia; 2 Institute of Technology, University of Tartu, Nooruse 1, 50411 Tartu, Estonia

**Keywords:** chromium oxide, thin films, atomic layer deposition, Ti-doped Cr_2_O_3_, mechanical properties, optical properties

## Abstract

Cr_2_O_3_ thin films were grown on a Si (1 0 0) substrate using Cr(thd)_3_ and O_3_ by atomic layer deposition (ALD) at substrate temperatures (*T_G_*) from 200 to 300 °C. X-ray amorphous films were deposited at a *T_G_* ≤ 225 °C, whereas at higher temperatures (*T_G_* ≥ 250 °C), the eskolaite phase was observed in the films. The growth rate of the films increased from 0.003 to 0.01 nm/cycle by increasing *T_G_* from 200 to 275 °C. The relatively low growth rate of Cr(thd)_3_—O_3_ makes it appropriate for the ALD of precisely controllable solid solution-type ternary-component thin films. The Ti-doped Cr_2_O_3_ film showed higher hardness (16.7 GPa) compared with that of the undoped film (12.8 GPa) with similar thickness. The band gap values of the pure Cr_2_O_3_ corresponding to the indirect transition model showed no dependence on *T_G_*; however, doping the Cr_2_O_3_ with Ti decreased its band gap energy value from 3.1 to 2.2 eV.

## 1. Introduction

Chromium oxide thin films are widely used for many purposes, such as protective coatings in tribological applications [1], gas sensors [2], solar energy absorbers [3], corrosion-resistant applications in semiconductors [4], magneto-electric random access memories and THz spin-orbitronic devices [5], and for transparent conducting oxide applications [6] because of its high hardness (from 18 to 29.7 GPa) and wear resistance [7], high melting point (~2435 °C) [8], good corrosion and chemical resistance [9], and magneto-electric [10] and catalytic properties [11]. These coatings are deposited using different methods such as sol–gel [12], electrodeposition [13], plasma spray pyrolysis [14], thermal evaporation [15], molecular beam epitaxy [16], magnetron sputtering [17], pulsed laser deposition [18], chemical vapor deposition [19], and atomic layer deposition (ALD) [20]. ALD has marked advantages due to the deposition of thin films with uniform thickness and ultra-low surface roughness on the surfaces of complex shapes, which enables control of the film composition and thickness at the sub-nanometer level [21,22].

In the ALD of Cr_2_O_3_, various inorganic and organometallic metal precursors, together with ozone, water, and hydrogen peroxide, have been used previously [20,23,24]. Chromyl chloride (CrO_2_Cl_2_), one of the previously often used precursors of chromium [20], is nowadays forbidden in manufacturing applications. Among the different oxidation states of Cr, the most stable and common states are trivalent (Cr^3+^) and hexavalent (Cr^6+^) chromium, whereas Cr^6+^ in CrO_2_Cl_2_ is mobile in the environment and is acutely toxic and mutagenic compared to Cr^3+^ [25,26]. Therefore, to have an environmentally friendly deposition process, chromium acetylacetonate (Cr(acac)_3_) and tris (2,2,6,6 tetramethyl heptane 3,5 dione) chromium (III) (Cr(thd)_3_) have been considered as chromium precursors. Mandol et al. [27] and Tripathi et al. [24] reported the ALD of Cr_2_O_3_ using Cr(acac)_3_ and O_3_. In our previous work, the possibility of using Cr(thd)_3_ and O_3_ as the precursors to deposit Cr_2_O_3_ seed layers was studied [28]. However, the details of the growth process of Cr_2_O_3_ thin films from these precursors have not yet been published.

One of the possibilities to modify the properties of the films and widen the window of the application is doping them with other compounds. For instance, it has been demonstrated that the hardness of Cr_2_O_3_ films can be increased from 27.3 to 40 GPa by doping them with Zr [29]. Al-doped chromium oxide coatings have shown higher optical absorption and good thermal stability [30]. Doping with Cu displayed an improvement in the transparency of Cr_2_O_3_ films in the visible range and a reduction in the optical band gap energy (*E_g_*) from 2.94 to 2.51 eV [31]. Adding chromium as a dopant element also changed the properties of the films; for instance, the visible-light photocatalytic activity of TiO_2_ films deposited by the hydrothermal method was improved by doping with Cr [32]. By doping Cr transition-metal ions into the ZnO matrix, the photoluminescence spectrum showed that UV emission radiation with a wavelength shorter than 385 nm was eliminated and the stability of the ZnO films increased under exposure to an oxygen atmosphere [33].

Among the variety of doping elements, titanium has been shown to improve the physical properties of Cr_2_O_3_ films. Room-temperature magnetron-sputtered corrosion-resistant Cr-Ti-O coatings exhibited hardness values up to 30.9 GPa [34]. A small concentration of titanium in Cr_2_O_3_ films can lead to large variations in the magnetically active oxygen vacancy defect states, making it a promising material for magneto-optic/magneto-electric applications [35]. The conductivity of Cr_2_O_3_ films can be changed from p-type to n-type by adding TiO_2_ because of the increase in the concentration of electrons [36]. Taking into account the number of changes that occur in the properties of Cr_2_O_3_ films by doping Ti, the number of reports and publications devoted to the analysis of the structural, mechanical, and optical properties of these ultra-thin films deposited by the ALD method is relatively small. This study aimed to present results about both ALD processes and the physical properties of pristine Cr_2_O_3_ and Ti-doped Cr_2_O_3_ thin films. The present research study contains two sections. The first section includes the results of the ALD of Cr_2_O_3_ using Cr(thd)_3_ and O_3_. The second section describes the effect of the Ti dopant element on the phase composition and the mechanical and optical properties of Cr_2_O_3_.

## 2. Materials and Methods

The Cr_2_O_3_ films were deposited on Si (100) substrates using an in-house-built flow-type ALD reactor [37] at temperatures (*T_G_*) from 200 °C to 300 °C. Before loading into the reactor, all samples were pretreated with a mixture of sulfuric acid and hydrogen peroxide and rinsed in deionized water, followed by etching in 5% HF solution and rinsing in deionized water again. Cr(thd)_3_ (99.99%, Strem Chemicals, Inc., Newburyport, MA, USA) preheated up to 106 °C and ozone (O_3_) at room temperature were used as Cr_2_O_3_ precursors. Nitrogen (99.999%, Linde Gas, Hong Kong) was used as a carrier gas. TiCl_4_ (99.9%, Aldrich, St. Louis, MO, USA) in reaction with O_3_ was used for doping Cr_2_O_3_ with Ti in the films. One ALD cycle of the Cr_2_O_3_ films included a series of exposure of Cr(thd)_3_ for 2 s; purge with N_2_ for 5 s; exposure of O_3_ for 1, 2, 3, or 5 s; and a second purge for 5 s, abbreviated in the following as 2/5/O_3_/5, where O_3_ denotes the exposure time of ozone that was preferentially equal with 2 s but was also varied from 1 to 5 s when investigating the effect of ozone exposure time on the growth process. The number of ALD cycles of pristine Cr_2_O_3_ films varied from 500 to 6000. The ALD super cycle of Ti-doped Cr_2_O_3_ films (CTO) was composed of a series of Cr_2_O_3_ cycles with the formula 5/2/2/5 repeated 15 or 30 times, followed by one exposure of TiCl_4_ for 2 s, purge for 2 s, exposure of O_3_ for 5 s, and purge for 5 s. The maximum number of super cycles was 80. The doped Cr_2_O_3_ films were grown at a *T_G_* of 275 °C. During the deposition, the total gas pressure in the reaction zone was maintained at about 2.2 mbar, and the nitrogen carrier gas flow rate was 230 sccm.

The elemental compositions of the deposited films were determined using an X-ray fluorescence (XRF) spectrometer ZSX400 (Rigaku, Tokyo, Japan). The phase composition of the films was characterized by the grazing incidence X-ray diffraction method (GIXRD, grazing angle *ω* = 0.5°). The thickness density and surface roughness of the films were characterized by the X-ray reflectivity method (XRR). The standard deviation for density values calculated by XRR was approximately 0.1 g/cm^3^. All GIXRD and XRR measurements were performed on an X-ray diffractometer SmartLab (Rigaku, Tokyo, Japan) using Cu Kα radiation (*λ* = 0.154178 nm, tube power 8.1 kW). The identification of Cr_2_O_3_ and TiO_2_ crystalline phases was based on the X-ray diffraction database PDF-2 (version 2023, International Centre for Diffraction Data, PA, USA). The X-ray apparent volume weighted crystallite size (*<D>_v_*) was calculated from Scherrer’s formula:

$${<D>}_{v}=\frac{\lambda }{\beta cos\theta }$$

where *λ* is the wavelength of the X-rays, *β* is the integral width of a diffraction reflection corrected by instrumental function based on standard reference material SRM-660 (LaB_6_), and *θ* is the Bragg angle of the reflection [38]. The texture of the films was characterized by calculating the texture coefficient (*TC*) [39].

The surface microstructure was characterized with a scanning electron microscope (SEM, FEI-Nova NanoSEM-450). The optical properties of the films were measured using a spectroscopic ellipsometer (SE) GES5E (Semilab Sopra), and data were analyzed using SEA software (Semilab Sopra). A mixture of the Tauc–Lorentz and Lorentz models was used to fit the measured data of films with a thickness of *t* ≥ 10 nm. The hardness and elastic modulus of the deposited films were investigated using the Hysitron Triboindenter TI 980 nanoindentation device (Bruker, Billerica, MA, USA). The samples were measured in continuous stiffness mode using a Berkovich-type diamond tip. The device was calibrated using a fused quartz standard. The strain rate of 0.05 nm/s and tip vibration frequency (220 Hz) were kept constant during the calibration and measurements. The calibrations were performed before and after the measurements. The overall arithmetic average of 16 measurements on the fused quartz standard showed a hardness of 9.2 ± 0.6 GPa and an elastic modulus of 70 ± 8 GPa at displacement range from 5 nm to 50 nm. A single indentation measurement consisted of sixty steps along the full displacement range, which depended on the actual thickness of the film and ranged up to 40 nm in the direction of the surface normal of the sample. The hardness and elastic modulus of the films were measured under a load of 250 μN at thirty different points on each sample. A few (maximum of five) measurements of hardness and modulus values falling considerably outside of the values of the remaining measurements were removed before averaging and calculating standard deviations. To minimize the effect of substrate on the hardness values of the films, the hardness values were determined at 10 nm of indent displacement.

## 3. Results and Discussions

### 3.1. The Growth of Cr_2_O_3_

**Figure 1:**
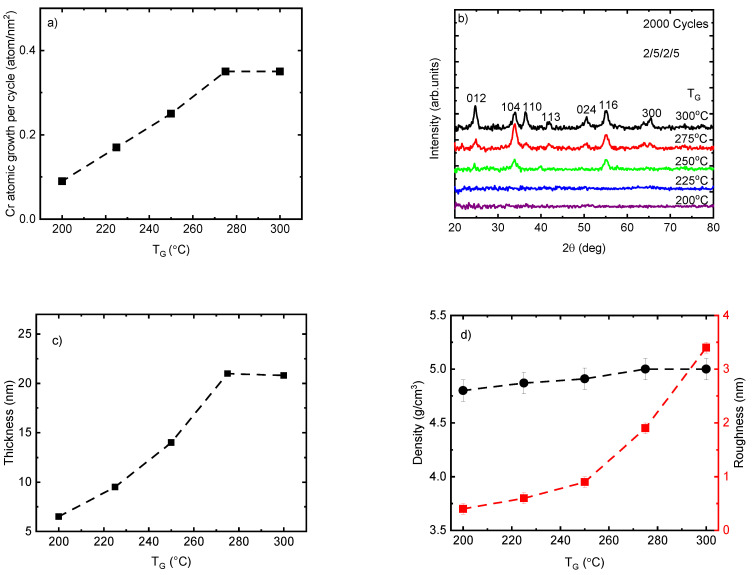
(**a**) Cr_2_O_3_ atomic growth rate per cycle; (**b**) GIXRD patterns showing the reflections indexed for the eskolaite phase; (**c**) thickness and (**d**) density and roughness of the Cr_2_O_3_ films at a variety of deposition temperatures. The label 2/5/2/5 relates to the exposure and purge duration time of Cr(thd)_3_ and ozone precursors.

To understand the correlation between the growth rate and *T_G_*, the Cr_2_O_3_ films were deposited using 2000 ALD cycles at *T_G_* from 200 to 300 °C. The Cr atomic growth of the films per cycle showed an increase almost linearly from 0.09 to 0.35 atom/nm^2^ (0.032 to 0.105 Å/cycle) when the *T_G_* was raised from 200 °C to 275 °C, which can be explained by the increase in precursor reactivity in this temperature range (Figure 1a). With the further increase of the *T_G_* to 300 °C no growth rate increase was observed if the samples positioned at the same place in side of the reactor are compared. However, at this *T_G_* there started to be a dependence of the films thickness to the distance from the precursor supply lines. In case, the growth rate for the films positioned 30 mm from the supply lines is lower than one obtained at 90 mm being 0.35 and 0.42 atom/nm^2^, respectively. Therefore, at this *T_G_* the films growth is not only limited by the ALD but some other parallel processes as well. The growth rate of Cr(thd)_3_ and O_3_ in this work is approximately three times lower than the corresponding values reported for Cr(acac)_3_ and ozone at *T_G_* from 150 to 300 °C [27]. Self-limited growth with a rate of 0.5–1 Å/cycle was also reported previously at *T_G_* of 330–420 °C for the ALD of Cr_2_O_3_ thin films using CrO_2_Cl_2_ and CH_3_OH [20]. The lower growth rate of Cr(thd)_3_—O_3_ in comparison with other precursors renders it a suitable candidate as a dopant material because of the feasibility of controlling the precise concentration of the doping. It is worth mentioning that, the lower growth rate of other volatile *β*-diketonate compounds such as Sc(thd)_3_ in combination with O_3_ in comparison with other precursors such as (C_5_H_5_)_3_Sc with H_2_O has been reported previously [40].

According to the GIXRD analysis, the deposited Cr_2_O_3_ films using 2000 ALD cycles at *T_G_* ≤ 225 °C did not show any diffraction reflections and therefore can be considered as X-ray amorphous (Figure 1b). It is important to note that these films (*T_G_* ≤ 225 °C) were very thin (*t* < 10 nm), and therefore the disappearing of diffraction reflections might be related also to the very low concentration of nanocrystalline phase that remains below the detection limit of the GIXRD method. The diffraction pattern of films showed a continuous increase in the number and intensity of diffraction reflections when the *T_G_* increased from 250 °C to 300 °C. The maximum number of reflections was observed on the diffraction pattern of film deposited at 300 °C. These seven peaks at 24.74°, 33.86°, 36.44°, 41.84°, 50.48°, 55.08°, and 65.26° belong to the eskolaite phase (α-Cr_2_O_3_) and can be indexed as 012, 104, 110, 113, 024, 116, and 300, respectively (PDF-2 database card # 00-038-1479). These changes in the composition of the films from X-ray amorphous to apparently crystalline are in correlation with the increase in growth rate (Figure 1a), thickness (Figure 1c), and roughness of the films (Figure 1d) by increasing *T_G_* from 200 to 300 °C. Remarkable changes in the relative intensity values of the reflections were observed at *T_G_* values from 275 to 300 °C (Figure 1b). These changes were quantitatively characterized by the *TC* calculated for the dominating reflections 012, 104, 110, and 116 (Table 1). At T_G_ = 275 °C, the *TC* was the highest for the 104 reflections and lowest for the 110 reflection, allowing to conclude that the growth at this *T_G_* (and probably also at lower *T_G_* values) proceeded preferentially in a crystallographic (104) plane or in a plane whose orientation is close to this plane—e.g., the (001) plane. When the *T_G_* was increased to 300 °C, the standard deviation over all *TC* values decreased considerably and the individual *TC* values approached the theoretical value calculated for the ideal powder sample (*TC* = 1.0 for all reflections). This was a clear indication that a more polycrystalline type of growth was observed when the T_G_ was increased up to 300 °C.

Interestingly, the density of the films increased only slightly from 4.8 to 5 g/cm^3^ within the whole range of observed *T_G_*. Considering the standard deviation (0.1 g/cm^3^), these values are close to the theoretical density of the eskolaite phase (5.2 g/cm^3^ (Figure 1d). This can be explained by the formation already at the lowest *T_G_* (≤225 °C) of a relatively high-density nanocrystalline structure composed of crystallites with mean sizes of 1–3 nm that generate diffraction reflections being too broad and weak for their registration even by GIXRD. The surface roughness of the Cr_2_O_3_ films was similar to the roughness value of the Si (100) up to 250 °C, being less than 1 nm, and increased to 3.4 nm by increasing *T_G_* up to 300 °C, which could be related to the changes in the film thickness and phase composition of the films from X-ray amorphous to textured and polycrystalline (Figure 1d).The increase in roughness values by increasing *T_G_* may contribute to an increase in the growth rate of the films by increasing the specific surface area of adsorption for precursor molecules.

**Figure 2:**
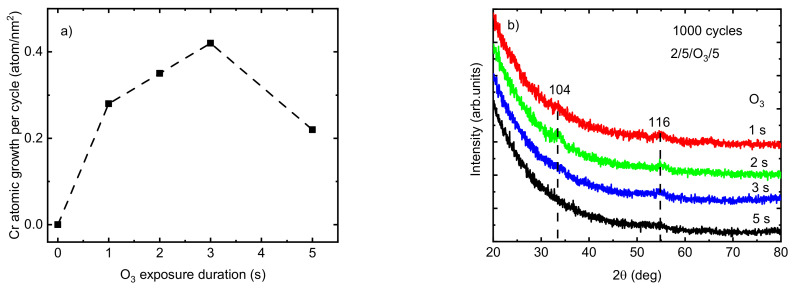
(**a**) The variation in the atomic growth rate of Cr_2_O_3_ deposited at T_G_ = 275 °C as a function of O_3_ exposure time; (**b**) GIXRD patterns at different O_3_ exposure times (the dotted vertical lines at the positions of indexed eskolaite reflections are guides for the eye).

Allowing sufficient precursor exposure time ensures the presence of enough precursors within the ALD reaction zone, which enables the reactions to continue until all existing surface sites have converted into new surface species and hinder any further continuation of the reaction. The effect of O_3_ exposure time on the growth rate of the Cr_2_O_3_ films deposited at *T_G_* = 275 °C was studied with a constant exposure duration of 2 s for Cr(thd)_3_ and a purging time of 5 s after both precursors (Figure 2a). An increase in Cr atomic growth per cycle was observed from 0.28 to 0.42 atom/nm^2^ by increasing the O_3_ exposure duration time from 1 to 3 s. However, the atomic growth rate of films decreased to 0.22 atom/nm^2^ when the O_3_ exposure time exceeded 3 s. The observed etching behavior at an O_3_ exposure duration of 5 s can be explained by the release of volatile or gaseous phase products [27,41]. Providing an excessive amount of ozone in the reaction zone may cause its interaction with adsorbed atomic oxygen species on the film surface, resulting in adsorbed peroxide groups and gaseous oxygen. Thereafter, these adsorbed peroxide groups decompose into molecular oxygen from the surface. Furthermore, an excessive amount of O_3_ in the reaction zone is known to lead to the formation of metal oxides with higher oxidation states such as CrO_3_, which is unstable and volatile at higher temperatures [27,41,42,43]. Similar results have been reported previously by Mandol et al. [27], who observed a decrease in the growth rate of Cr_2_O_3_ films by increasing the O_3_ exposure time to over 2 s. Etching is a well-known phenomenon observed during the ALD process of different precursor combinations. For instance, the etching of tantalum oxide film in a continuous TaCl_4_ flow as a result of the desorption of oxychlorides was reported previously by Aarik et al. [41]. It is desirable for producing thin films with a smoother surface and pattern-transfer applications in semiconductor production and the fabrication of nano-devices [44,45]. For instance, the roughness of GaN films was decreased from 0.8 to 0.6 by atomic layer etching [46]. However, etching has to be avoided when it prohibits thin film growth. Using an O_3_ pulse duration of 1 to 3 s (*T_G_* = 275 °C), the films showed weak reflections relating to the eskolaite phase at 33.86° and 55.08°, indexed as 104 and 116, respectively (PDF-2 database card # 00-038-1479). The film was X-ray amorphous when the O_3_ pulse duration was 5 s (Figure 2b).

**Figure 3:**
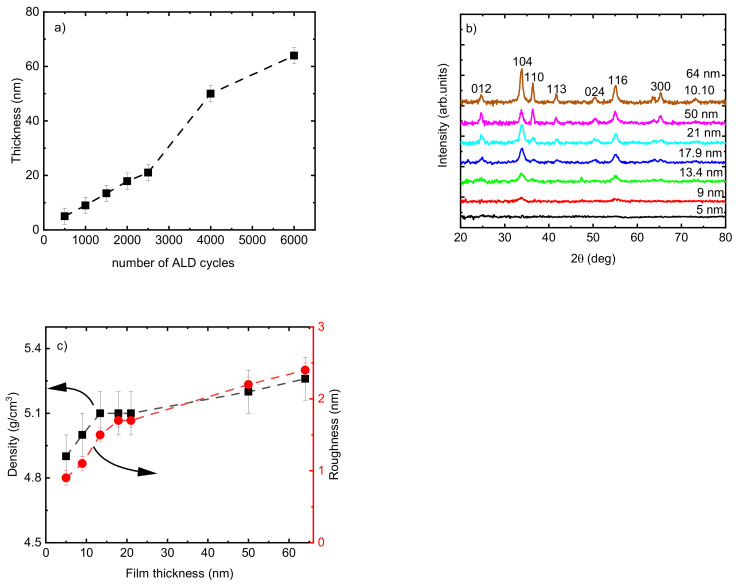
(**a**) Thickness of Cr_2_O_3_ films following a varied number of ALD cycles; (**b**) GIXRD patterns; (**c**) density and roughness of the Cr_2_O_3_ films as a function of film thickness. The films were deposited at *T_G_* = 275 °C.

The thickness of the Cr_2_O_3_ films changed linearly from 5 to 21 nm by increasing the number of ALD cycles from 500 to 2000 for films deposited at *T_G_* = 275 °C (Figure 3a). This linear increase was not followed with the same slope when the number of ALD cycles further increased to 4000 (Figure 3a). The microstructure of the films changed from X-ray amorphous film with a thickness of 5 nm to polycrystalline by increasing the thickness up to 64 nm (Figure 3b). By increasing the thickness of the films from 21 to 64 nm, the films exhibited a more polycrystalline character of growth (more reflections appearing from atomic planes with quite different mutual orientations, e.g., 110, 012, and 300). The calculation of *TC* showed changes in the preferred growth orientation of the films from the (104) plane to the (116) plane when the thickness of the film increased from 21 nm to 50 nm. The highest *TC* value also was achieved for 104 reflection for the film of 64 nm thickness, which confirms that the growth proceeded preferentially in the (104) plane. Deposition of Cr_2_O_3_ films with eskolaite structure and preferred growth orientation in the (104) plane was reported previously [47].

**Figure 4:**
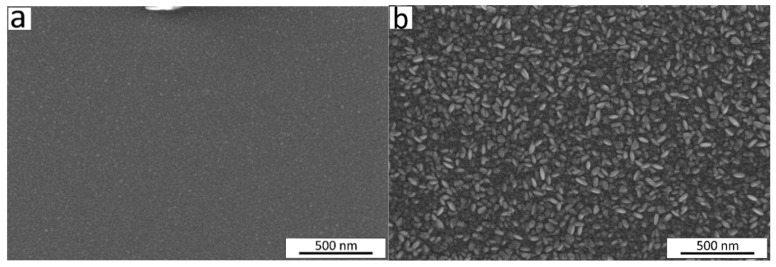
SEM images of the Cr_2_O_3_ films deposited at 275 °C with thicknesses of (**a**) 21 nm and (**b**) 50 nm.

This change in crystalline fraction in the films when increasing *t* from 5 to 64 nm was accompanied by an observed increase in density from 4.9 to 5.2 g/cm^3^ and in roughness from 0.9 to 3.4 nm (Figure 3c). The range of thicknesses from 21 nm to 50 nm showed the highest slope of the function *t* = *f* (number of cycles) (Figure 3a), and the surface morphology of the films exhibited remarkable changes as seen from the scanning electron microscope (SEM) images (Figure 4). The average size of the grains shown on the SEM image for the film with *t* = 21 nm was approximately 12 nm. The 2.4 times thicker film (*t* = 50 nm) exhibited grains with asymmetric shapes, having mean sizes of 23 nm and 50 nm in two perpendicular directions. It can be predicted that the growth of the film proceeds in different stages—starting with a preferential orientation type up to the thickness of approximately 20 nm, followed by the relatively intensive formation of crystallites with asymmetric shapes in the range of *t* = 20–50 nm, and finishing with the enlargement of the already grown crystallites and possibly also with the formation of new crystallites whose orientation is determined by the presence and orientation of the underlying crystallites. An increase in crystallite size by increasing the film thickness for Cr_2_O_3_ films using CrO_2_Cl_2_ and CH_3_OH was reported previously [48].

### 3.2. The Growth of CTO Films

**Figure 5:**
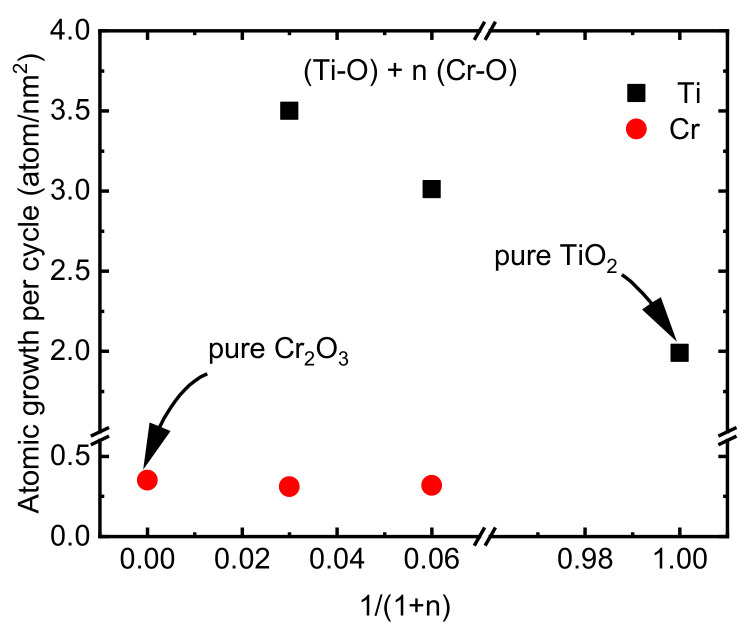
Growth rates of Ti and Cr atoms in a varying relative number of Cr—O precursor cycles.

The deposition formula, chemical composition, and thickness of the CTO films deposited on the Si substrate using ALD can be observed in Table 2. The lower growth rate of Cr(thd)_3_ and O_3_ that was discussed above (Section 3.1) can make it a good candidate for doping other materials with low concentrations of Cr. However, in the current study, since a very low amount of Cr (maximum 1 atm%) caused no remarkable change in the microstructure and properties of the TiO_2_ films, we focused mostly on films with Ti/(Ti+Cr) ratios of 0.25 and 0.45. The film growth rate was studied as the surface density of Cr and Ti atoms deposited per one ALD super cycle (Figure 5). The deposition density of Ti atoms was approximately 1.5–1.8 times higher on Cr_2_O_3_ film compared to depositing on binary TiO_2_ films. These changes in deposition density can be explained by differences in the adsorption properties of Cr_2_O_3_ and TiO_2_ surfaces. A higher growth rate of TiO_2_ on RuO_2_ in comparison with its growth rate on a Si substrate using TiCl_4_ and O_3_ as precursors was reported previously due to the excessive oxygen present on the RuO_2_ surface compared to the TiO_2_ surface [49]. Unlike chlorine-free pure Cr_2_O_3_ films, the doped films contained a small amount of chlorine (0.06 to 0.03 atomic%) which was lower than the amount of Cl in the pure TiO_2_ film (0.08 atomic%) or in the TiO_2_ films deposited previously on the Si substrate using TiCl_4_ and O_3_ at 300 °C [49].

**Figure 6:**
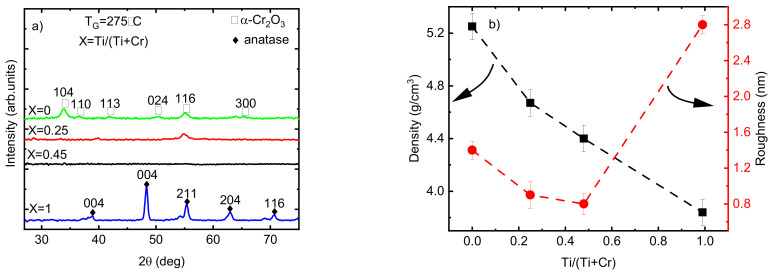
(**a**) GIXRD patterns; (**b**) density and roughness values of the Ti-doped Cr_2_O_3_ films as a function of Ti atomic ratio.

According to the GIXRD analysis, the phase composition of the Cr_2_O_3_ films changed from crystalline to X-ray amorphous by increasing the Ti/(Ti+Cr) atomic ratio from 0 to 0.45 (Figure 6a). Increasing the Ti amount in the films decreased the crystalline fraction of the films, observed through the disappearance of most of the diffraction reflections (except the 116 reflection for the film with Ti/(Ti+Cr) = 0.25). This may be attributed to the negative effect of Ti atoms on the nucleation of crystallites with an eskolaite structure. The difference between the atomic radii of Cr^3+^ and Ti^4+^ is small (0.62 Å compared to 0.68 Å, respectively); however, the inclusion of two Ti^4+^ ions into the lattice of eskolaite requires, according to the charge balance, one interstitial oxygen, which makes the formation of the eskolaite phase unfavorable.

Remarkable changes in the density and roughness of the films when varying the relative concentration of Ti and Cr atoms were observed (Figure 6b). The decrease in the roughness of the films by increasing the atomic ratio of Ti from 0 to 0.45 is directly related to the appearance of the X-ray amorphous phase and a decrease in the crystallite size of the films. The density of Cr_2_O_3_ films decreased from 5.2 g/cm^3^ for pristine Cr_2_O_3_ film to 4.7 and 4.4 g/cm^3^ by increasing the atomic ratio of Ti to 0.25 and 0.45, respectively (Figure 6b). The decrease in the density of doped films can be related to the lower density of anatase (4.2 g/cm^3^) compared to that of eskolaite (5.2 g/cm^3^) as well as the lower density of X-ray amorphous phase compared with the density of eskolaite or/and anatase phases.

### 3.3. Mechanical Properties of the Films

**Figure 7:**
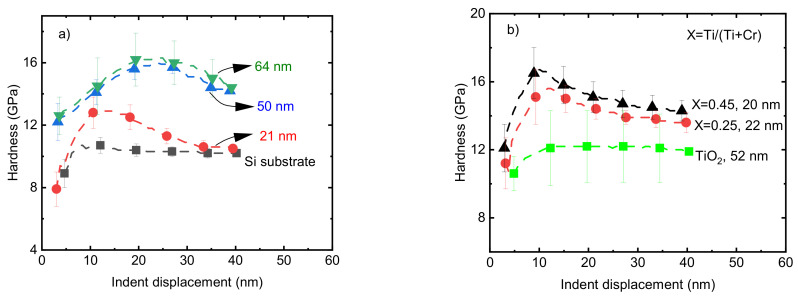
The hardness of (**a**) pristine Cr_2_O_3_ films with different thicknesses and (**b**) Ti-doped Cr_2_O_3_ films with different Ti atomic ratios. The thickness values of films are shown at the legend of the corresponding curves.

The hardness values of pristine Cr_2_O_3_ films on a Si substrate with *t* = 21, 50, and 64 nm were 12.8 ± 0.7, 14 ± 0.9, and 14.5 ± 0.9 GPa, respectively, at an indent displacement of 10 nm (Figure 7a). The dependence of the film hardness on the thickness of the films could be due to the effect of the low hardness of the softer substrate, since in the range of nanometer-scale thickness, the effect of the mechanical properties of the substrate cannot be ignored [50,51]. Previously, a 74 nm thick Cr_2_O_3_ film deposited from CrO_2_Cl_2_ and CH_3_OH precursors showed a hardness of 15.5 GPa [20]. Taking into account the standard deviations, the reported value is comparable with the hardness of the pristine Cr_2_O_3_ films with a thickness of 64 nm in the current work. Ti-doped Cr_2_O_3_ films with Ti/(Ti+Cr) atomic ratios of 0.25 and 0.45 in the range of *t* = 20–22 nm showed hardness values of 15.2 ± 1 and 16.7 ± 1 GPa, respectively, which are approximately 3–4 GPa higher compared to the hardness of the pristine Cr_2_O_3_ film with approximately the same thickness (Figure 7b).

Increases and decreases in mechanical properties are frequently correlated with changes in the density and nanostructure of films. The changes in the nanostructure of the films could be recognized by the calculation of crystallite size using X-ray diffraction patterns. The crystallite sizes of both pure Cr_2_O_3_ and those CTO films, with a Ti atomic ratio of 0.25, determined using 116 reflection of eskolaite, showed a reduction in crystallite size from 13 nm to 9 nm upon doping with Ti. This decrease in crystallite size was accompanied by a corresponding decrease in the crystalline fraction (Figure 6a) and roughness of the films (Figure 6b) upon Ti doping. The decrease in crystallite size could be one of the reasons for the higher hardness values of the Ti-doped Cr_2_O_3_ films [52]. The increase in hardness values may also be due to the formation of an amorphous/crystalline nanocomposite structure and the presence of amorphous TiO_2_ between the nanocrystallites of the eskolaite phase. In this mechanism, a combination of the absence of dislocation activity in the small nanocrystals, the grain boundary sliding blocking by the formation of a strong interface between the amorphous and crystalline phases, and crack propagation blocking in the amorphous phase by the presence of nanocrystallites has been shown to lead to a hardness increase in films [53,54]. The effect of changes in nanostructure on the mechanical properties of chromium-containing ternary films such as Cr-V-O and Cr-Ti-O has been shown previously [55,56]. A hardness of ~16 GPa was reported for Cr_2_O_3_-TiO_2_ composite coatings with 16 atomic% of TiO_2_ [56]. The variations in hardness are correlated with the density of the films, and frequently, higher hardness values have been observed for films with higher density values [57,58]. However, in the current study, the results do not follow the same trend after doping Cr_2_O_3_ films with Ti (Figure 6b and Figure 7b). The decrease in the density of Cr_2_O_3_ films after doping with Ti is most probably related to the difference in the densities of α-Cr_2_O_3_ (~5.23 g/cm^3^, ICDD PDF-2 card no. 00-038-1479) and TiO_2_ anatase (3.96 g/cm^3^, ICDD PDF-2 # 01-075-2552) and also the lower density of the X-ray amorphous phase compared with the density of eskolaite or/and anatase phases.

### 3.4. Optical Properties of Films

**Figure 8:**
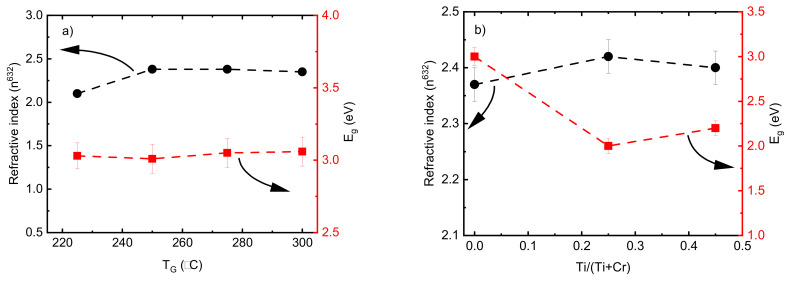
Refractive index and band gap energy values of (**a**) pristine Cr_2_O_3_ films as a function of deposition temperature and (**b**) doped Cr_2_O_3_ films as a function of Ti/(Ti+Cr) atomic ratio.

The refractive index values (*n*) of pristine Cr_2_O_3_ deposited by 2000 ALD cycles (thickness in Figure 1c) showed an increase from 2.15 to 2.35 when the *T_G_* increased from 225 to 250 °C and remained constant by increasing the *T_G_* up to 300 °C (Figure 8a). A low refractive index value of 1.43 was achieved for the film deposited at 200 °C using the same model; however, different refractive index values could be achieved by changing the model parameters. In this connection, an abnormally low refractive index value below 1.5 for HfO_2_ films with a thickness of ~5 nm was reported by Kasikov et al. [59]. The band gap energy value of the films were calculated using an indirect transition model [60] in an energy range from 2.5 to 4.5 eV. The *E_g_* values of the pristine Cr_2_O_3_ films were between 3.1-3.2 and no dependence on the *T_G_* was observed in the range of 225 to 300 °C (Figure 8a). These values are in good agreement with the *E_g_* values of Cr_2_O_3_ films reported recently [31]. According to earlier research works [31,61], changes in the optical properties are expected if the Cr_2_O_3_ films are doped with Ti. The refractive index value of the pure TiO_2_ was 2.50 ± 0.03, which is comparable with those results reported previously [62]. A decrease in *E_g_* values from 3.1 to 2.2 corresponding to the indirect transition model was observed by adding the Ti dopant element (Figure 8b). Similar results showing a reduction in *E_g_* for doped Cr_2_O_3_ films have been demonstrated previously. Zekaik et al. showed a reduction in *E_g_* from 2.94 eV for pure Cr_2_O_3_ films to 2.51 eV for Cu (12%)-doped Cr_2_O_3_ films [31]. They discussed that the decrease in *E_g_* values by increasing the doping element concentration could be related to the average bond energy, which is a function of the composition of films [31]. The reduction in the *E_g_* value by adding Ti may also be due to the formation of an impurity-like band in the Cr_2_O_3_ host. Similar behavior was reported previously when the *E_g_* of pristine Cr_2_O_3_ nanoparticles decreased from 2.85 to 2.75 eV with Ni doping [58].

## 4. Conclusions

This study focused on the ALD and characterization of Cr_2_O_3_ films using Cr(thd)_3_ and O_3_. The Cr atomic growth increased from 0.09 to 0.35 atom/nm^2^ with increasing temperatures (*T_G_*) from 200 °C to 275 °C followed by phase transformation from X-ray amorphous to crystalline containing an eskolaite phase, which was in correlation with the increase in growth rate, thickness, and roughness of the films. At 275 °C, etching took place when the O_3_ exposure duration exceeded 3 s due to the dissociative desorption of O_3_ on the metal oxide surface and the formation of volatile metal oxides. The microstructure of the films changed from X-ray amorphous to polycrystalline on increasing the thickness from 5 to 64 nm, which was accompanied by an increase in density from 4.9 to 5.2 g/cm^3^ and in roughness from 0.9 to 3.4 nm. The band gap values of the Cr_2_O_3_ corresponding to the indirect transition model showed no dependence on *T_G_*. The low deposition rate of the Cr_2_O_3_ films using Cr(thd)_3_ and O_3_ showed the possibility of precise doping of TiO_2_ films with a small amount of Cr (even ≤1 atm%). The doping of the Cr_2_O_3_ films with Ti influenced their properties significantly. Films with Ti/(Ti+Cr) atomic ratios of 0.25 and 0.45 exhibited a decrease in crystalline fraction, density, and roughness but an increase in hardness. This increase was attributed to changes in the nanostructure, including crystallite size reduction and the potential formation of an amorphous/crystalline nanocomposite structure. Doping of Cr_2_O_3_ with Ti changed the refractive indices from 2.35 to 2.45 and band gap energy from 3.1 to 2.2 eV in case of indirect transition model, while the Ti/(Ti+Cr) atomic ratio increased up to 0.45 due to impurity-like band formation and composition effects. The results contribute to a better understanding of the ALD of Cr_2_O_3_ films using Cr(thd)_3_ and O_3_ and the relationship between deposition parameters and Cr_2_O_3_ film properties, which can be valuable for optimizing the deposition of Cr_2_O_3_ films and offering insights into designing functional thin films with tailored properties.

## Figures and Tables

**Table 1:** Texture coefficient (*TC*) values for dominating reflections of Cr_2_O_3_ films deposited at two selected *T_G_* values. STD is the standard deviation calculated over all *TC* values of the reflections.

hkl	275 °C	300 °C
012	0.9 ± 0.09	1.4 ± 0.14
104	1.6 ± 0.16	0.7 ± 0.07
110	0.3 ± 0.03	0.8 ± 0.08
116	1.0 ± 0.1	0.9 ± 0.1
STD	0.46	0.27

**Table 2:** Characteristics of the ALD process and the CTO films grown at a substrate temperature of 275 °C.

ALD Formula	Number of Super Cycles	Film Thickness (nm)	Ti (atm%)	Cr (atm%)	O (atm%)	Cl (atm%)	Ti/(Ti+Cr)
Cr_2_O_3_	2000	18	0	42	58	0.0	0
(Cr—O) + (Ti—O)	500	33	32	1	66	0.04	0.97
(Cr—O) + 5(Ti—O)	140	38	33	0.2	66	0.06	0.99
(Cr—O) + 30 (Ti—O)	137	48	34	0	65	0.06	1
15 (Cr—O) + (Ti—O)	80	20	19	20	61	0.04	0.45
30 (Cr—O) + (Ti—O)	45	22	10	29	60	0.03	0.25
TiO_2_	1000	52	33	0	65	0.08	1

## Data Availability

The data presented in this study are available on request from the corresponding author.
